# Rheological and Structural Study of Salmon Gelatin with Controlled Molecular Weight

**DOI:** 10.3390/polym12071587

**Published:** 2020-07-17

**Authors:** Javier Enrione, Cielo Char, Marzena Pepczynska, Cristina Padilla, Adrian González-Muñoz, Yusser Olguín, Claudia Quinzio, Laura Iturriaga, Paulo Díaz-Calderón

**Affiliations:** 1 Biopolymer Research & Engineering Laboratory (BIOPREL), Escuela de Nutrición y Dietética, Facultad de Medicina, Universidad de Los Andes, Santiago 7620001, Chile; jenrione@uandes.cl (J.E.); mpepczynska@uandes.cl (M.P.); cpadillar@uandes.cl (C.P.); adrian.gonzalez@miuandes.cl (A.G.-M.); 2 Centro de Investigación e Innovación Biomédica, Facultad de Medicina, Universidad de los Andes, Santiago 7620001, Chile; 3 Departamento de Ciencias de los Alimentos y Tecnología Química, Facultad de Ciencias Químicas y Farmacéuticas, Universidad de Chile, Santiago 8380494, Chile; cchar@ciq.uchile.cl; 4 Centro Científico Tecnoloógico de Valparaíso (CCTVal), Universidad Técnica Federico Santa María, Valparaíso 2390123, Chile; yusser77@gmail.com; 5 Centro de Biotecnología (CB-DAL), Universidad Técnica Federico Santa María, Valparaíso 2390123, Chile; 6 Centro de Investigación en Biofísica Aplicada y Alimentos (CIBAAL), CONICET-Universidad Nacional de Santiago del Estero, Santiago del Estero 4200, Argentina; cmquinzio@hotmail.com.ar (C.Q.); laura.iturriaga@gmail.com (L.I.)

**Keywords:** salmon gelatin, molecular weight, helical structure, viscoelasticity

## Abstract

This study explores the molecular structuring of salmon gelatin (SG) with controlled molecular weight produced from salmon skin, and its relationship with its thermal and rheological properties. SG was produced under different pH conditions to produce samples with well-defined high (SGH), medium (SGM), and low (SGL) molecular weight. These samples were characterized in terms of their molecular weight (MW, capillary viscometry), molecular weight distribution (electrophoresis), amino acid profile, and Raman spectroscopy. These results were correlated with thermal (gelation energy) and rheological properties. SGH presented the higher MW (173 kDa) whereas SGL showed shorter gelatin polymer chains (MW < 65 kDa). Raman spectra and gelation energy suggest that amount of helical structures in gelatin is dependent on the molecular weight, which was well reflected by the higher viscosity and G′ values for SGH. Interestingly, for all the molecular weight and molecular configuration tested, SG behaved as a strong gel (tan δ < 1), despite its low viscosity and low gelation temperature (3–10 °C). Hence, the molecular structuring of SG reflected directly on the thermal and viscosity properties, but not in terms of the viscoelastic strength of gelatin produced. These results give new insights about the relationship among structural features and macromolecular properties (thermal and rheological), which is relevant to design a low viscosity biomaterial with tailored properties for specific applications.

## 1. Introduction

Many tons of fish by-products are generated by the fishing industry globally each year, including bones, guts, heads, tails, scales, and substantial quantities of skin. Fish skin is known to be a good source of collagen and gelatin [1,2,3]. Gelatin, a partially hydrolyzed form of the fibrillar protein of collagen, can be used for the design of multifunctional biomaterials and composites with interesting applications in the food [4], pharmaceutical, and biomedical industries [5,6,7,8,9,10].

Gelatin from fish adapted to cold water, such as those obtained from salmon skin and other species, shows lower gelling temperatures (<15 °C) compared to mammal and warm water fish gelatins [11]. Indeed, in the case of salmon gelatin (SG), it can to flow at room temperature even at high concentrations as its gelling temperature is close to 4 °C [12]. This property is explained by lower concentration of the imino acids proline and hydroxyproline, which promote the triple helix formation and physical folding kinetics from gelatin polymer strands upon cooling [13]. In addition, the lower viscosity has also been explained by the lower molecular weight of the salmon gelatin polymeric chains as shown by different analytical techniques [12,14]. Thus, the combined effect of varying imino acids content and molecular weight contributes to SG showing lower gel strength and different viscoelasticity than mammal gelatins.

Depending on the application where the SG could be used, its properties can be advantageous allowing the development of different strategies to design salmon gelatin-based biomaterials with tailored and engineered functional and technological properties. Indeed, low viscosity gelatins have been successfully used in applications that require small-scale constructs with well-defined geometries and tuned structures such as electrospinning [15], microfluidics, and micropatterning [16,17]. Other applications include sprayable food edible coatings [4], polymeric scaffolds, and more recently, bio-inks for 3D printing for tissue engineering applications [1,18,19]. These examples clearly show some of the advantages in using cold water fish gelatins compared to gelatins from mammal, enabled by its rheological properties, either to the design of novel product or the complex manufacturing processes. Warm blood mammal gelatins normally require higher working temperature conditions and lower concentrations to avoid clotting [20].

Despite these recent advances in research on SG, limited information is available about its physical properties and functionality when the polymer features well-defined molecular weights produced by various processing conditions. Only recently Diaz-Calderón [5] reported the effect of extraction conditions (pH and time) on the structure and physical properties of SG, showing the direct correlation among extraction conditions and biochemical profile, molecular weight, mechanical response, and crystallinity of gelatin. However, that study did not explore in the molecular configuration and rheological properties of SG. Thus, this type of studies are necessary because of the interesting perspectives of using salmon gelatin in the technological applications before explained, but also because SG is obtained from the skin of cultivated salmon specimens produced by sea farms where the quality and traceability are extremely well-controlled. Hence, is worth to mention that raw skins and therefore the gelatin obtained from them are characterized by their good and consistent quality and properties over time.

The purpose of this study is therefore to further explore the molecular structuring of salmon gelatin with controlled molecular weight, with the aim to improve the understanding of its effects on macroscopic features such as thermal and rheological properties of this low viscosity underutilized biomaterial. We believe that this information is necessary for a better understanding of the structure–function relationship for the rational design of SG bases materials with tunable properties at the macro scale required for applications with increasing level of complexity.

## 2. Materials and Methods

### 2.1. Salmon Gelatin Samples

SG samples with controlled molecular weight were obtained through an extraction method at different pHs from skins from Atlantic salmon (*Salmo salar*). Salmon skins were provided by a local salmon processing company and stored at −40 °C until further use. Prior the extraction process, the skins were manually cleaned using a sharp knife in order to eliminate all residues of muscle and scales, and then were cut into squared pieces (3 cm **×** 3 cm). The extraction protocol followed the methodology proposed by Hou and Regenstein [21] with some modifications [14]. Briefly, alkaline pre-treatments were carried out by submerging the chopped skins in NaOH 0.1 M at 10 °C for 1 h. Water processed by reverse osmosis (conductivity < 10 μS) was used for preparing the solutions. This pre-treatment was repeated twice. Then, the processed skins were washed and treated with a 0.05 M acetic acid solution at 10 °C for 1 h. After rinsing, the gelatin extraction process was carried out under different pH conditions (3, 4, and 5 adjusted using acetic acid and NaOH) at 60 °C for 3.5 h. The supernatant was subsequently vacuum filtered and dried in a convective oven (Wiseven, Seoul, Korea) at 60 °C for 48–72 h as required. The dried gelatin was colorless and free from fishy odor. The produced gelatin was then ground (Knifetec, FOSS Analytical Co. Ltda, Hillerod, Denmark) and stored at 5 °C until further use. The salmon gelatin samples were identified as SGL (low molecular weight), SGM (medium molecular weight), and SGH (high molecular weight) for gelatin produced at pH 3, pH 4, and pH 5, respectively.

### 2.2. Proximate Composition

Proximate composition of all three SG samples was assessed according to the methods described by AOAC [22] as follow: moisture content by oven drying at 105 °C for 24 h, fat content by Soxhlet, protein content by the Kjeldhal (%N × 5.55) and ash content by using a muffle furnace at 550 °C. Non-nitrogenous fraction was determined by the weight difference, which was calculated by subtracting to the 100% the total of other components (in percentage) present in samples [5].

### 2.3. Molecular Weight by Capillary Viscometry and Electrophoresis

The average molecular weight (MW) of the polymer chains present in each SG sample was determined by capillary viscometry [12,23]. This method considers that at infinite dilution condition (*C* → 0), the reduced viscosity (*ƞ*_red_) and the inherent viscosity (*ƞ*_inh_) of a solution are defined as the intrinsic viscosity [*ƞ*], which can be correlated with the MW through a mathematical model described by the Mark-Houwink-Kuhn-Sakurada (MHKS) equation [23]:

[*ƞ*] = *K* × MW*^a^* (1)

where *K* and *a* are constants dependent on the nature of the solvent and the polymer conformation.

The gelatin samples were diluted in a 0.1 M NaCl solution, at concentrations ranging from 0 to 6 g L^−1^ and left overnight at 4 °C for complete hydration. The flow time of the gelatin suspensions was measured using an “Ostwald” viscometer (“Q” Glass Company Inc., Towaco, NJ, USA) immersed in a thermoregulated water bath set at 50 °C, following the procedure described by Enrione et al. [24] and Quero et al. [12]. Viscometer of size 50 was used for testing SGL and SGM, whereas viscometer size 75 was used for gelatin SGH, according with the higher viscosity of SGH. The *η*_red_ and *η_i_*_nh_ viscosities were plotted against the gelatin and NaCl solutions and the limit near zero concentration (*C* → 0) was taken as the intrinsic viscosity [*η*]. The determination of MW of the produced salmon gelatin was carried out using the values of *a* and *K* reported by Quero et al. [12].

Complementary to this technique, the molecular weight (MW) distribution of the SG polymer chains was determined by sodium dodecyl sulfate polyacrylamide gel electrophoresis (SDS-PAGE). All the steps for the SDS-PAGE were performed as described before by Díaz-Calderón et al. [5] using 4–15% acrylamide gradient precast gels (Bio-Rad Laboratories, Inc., Irvine, CA, USA). All samples were heated at 95 °C for 5 min before loading (67 μg of gelatin) and a standard molecular weight marker in the 10–250 kDa range was used (KaleidoscopeTM, Precision Plus Protein StandardsTM, Biorad). The electrophoresis was run at 100 V and the resulting gel was stained with 0.25% Commassie blue R250.

### 2.4. Amino Acidic Profile by HPLC

The concentration of the amino acids present in SG samples was determined by reverse-phase high-performance liquid chromatography (RP-HPLC) following the methodology described by Rebane and Herodes [25] with the modifications reported by Díaz-Calderón et al. [5]. The amino acids were separated and quantified using a chromatograph (Waters 600 controller, Milford, MA, USA) with a diode array detector (Waters 996) using a RP18 column (150 mm × 4.6 mm, particle size 5 μm) (Luna, Phenomenex, Los Angeles, CA, USA). Amino acid content was reported as g/100 g of protein.

### 2.5. Isoelectric Point by Zeta Potential

The isoelectric point (pI) of the SG suspensions (0.015% w/v) was determined by assessing changes in the zeta potential at various pH values using a Zetasizer Nano-Z instrument (Malvern Panalytical Ltd., Westborough, MA, USA). The pH was automatically adjusted from pH 3 to pH 12 by an automatic titrator attached to the instrument (Malvern Panalytical Ltd., Westborough, MA, USA). Two independent runs with triplicate measurements were performed (n = 6).

### 2.6. Molecular Configuration by Raman Spectroscopy

For this analysis, SG suspensions at a concentration of 7% w/v were prepared at 60 °C for 40 min, which were then poured onto Teflon molds and maintained at 5 ± 0.5 °C for 7 days in order to obtain flat and transparent films with a final thickness of ~0.3 mm. The chemical groups vibrations and molecular configuration of the polymer were analyzed using a Raman spectrometer (XploRA PLUS, Horiba Scientific, Lille, France) equipped with a near infrared laser (λ 785 nm and 1 μm diameter). In order to avoid sample burning, the power of the laser was set to 70 mW. All spectra were acquired using a diffraction grating with a groove density of 600 gmm^−1^. The laser was focused on the sample’s surface using an optical microscope (Olympus BX41) using a ×100 long-working distance objective (PL Fluotar, NA = 0.55). Each spectrum was acquired within the wavenumber range of 200–3500 cm^−1^ using an exposure time of 20 s and three accumulations. All spectra were normalized and corrected using the instrument software (LabSpec 6 software version 6.4, Horiba Scientific, Lille, France). Five replicates were used for each measurement.

### 2.7. Thermal Properties by DSC

SG suspensions (10% w/v) were prepared in distilled water under stirring at 60 °C for 40 min. The samples (70 μL) were loaded into aluminum DSC pans (100 μL), hermetically sealed and subjected to thermal scans using a DSC-1 instrument (Mettler-Toledo, Greinfensee, Switzerland). Prior to the measurements, the DSC was calibrated using indium (melting temperature 156.6 ± 1.56 °C and melting enthalpy ΔH = 28.6 ± 1 J g^−1^). An empty pan was used as reference. All experiments followed the protocol of cooling down the samples from 25 °C to −10 °C at 2 °C min^−1^. Measurements were carried out using six replicates. The gelling temperature (T_gelling_) of all SG samples was determined from the onset of the exothermic peak observed on the cooling scans. The energy associated to the coil to helix transitions (gelation energy) was defined as the change in enthalpy (ΔH) and it was calculated from the area under the exotherm curve and expressed based on the dry mass of gelatin. The weight of all pans was checked before and after the measurements to ascertain that no water loss occurred during analysis.

### 2.8. Viscoelastic Behavior by Rheology

Viscoelastic properties of the SG suspensions (10% w/v) were measured using a rheometer (Discovery HR2, TA Instruments, New Castle, DE, USA). Flow curve measurements were carried out using a cone geometry (stainless steel, 40 mm diameter, 0:30:7 angle, and 15 μm truncation), whereas oscillatory tests were performed using a flat plate geometry (stainless steel, 50 mm diameter). During analysis, the samples were covered with a solvent trap to avoid water evaporation. Data analysis was conducted using the TRIOS software package (TA Instruments, New Castle, DE, USA). All analysis was carried out using at least five replicates.

#### 2.8.1. Steady-Shear Flow Measurements

The flow behavior of the SG samples produced was determined at 25 °C. The shear rate range used was 0.01–1000 s^−1^. The range of linear viscosity values for each gelatin was obtained from the viscosity **vs.** shear rate plot.

#### 2.8.2. Temperature Sweep Test

*Steady shear curves:* The apparent viscosity of the gelatin suspensions was measured as a function of temperature from 40 °C to 0 °C, with cooling rate of 2 °C min^−1^ and shear rate of 40 s^−1^. The flow temperature ramps were plotted as apparent viscosity **vs.** temperature.

*Dynamic curves:* The viscoelastic behavior of the SG suspensions was measured under oscillatory at 1 Hz and 2% strain within the linear viscoelastic range (LVR) at all temperatures measured. The samples were cooled from 40 to 0 °C at 2 °C min^−1^, and the rheological parameters elastic modulus (G′), loss modulus (G′′), and tan δ (G′′/G′) were determined. Gelling temperature (T_gelling_) was associated to the G′′ and G′ intersecting values upon the cooling step.

*Frequency sweep test:* Each SG suspension was cooled at 2 °C min^−1^ from 40 °C to 5 °C below the gelling temperature obtained in previous section. Then, the temperature was held constant and the response of the SG moduli (G′, G” and tan δ) to increasing frequency (0.1 to 100 Hz) at a strain 2% was assessed.

### 2.9. Statistical Analysis

When appropriate, one-way analysis of variance (ANOVA) and Tukey test with a 95% confidence level were carried out to statistically assess significant differences (*p* ≤ 0.05) among the gelatins extracted under different pH conditions. These analyses were conducted using the software Statgraphics Centurion XVI (Statgraphics Technologies Inc., The Plains, VA, USA).

## 3. Results and Discussions

### 3.1. Proximate Composition

The proximate composition of the SG samples with controlled molecular weight (SGL, SGM, and SGH), which were obtained through an extraction method at different pH values, is presented in Table 1. For all the SG samples at least ~86% corresponded to protein and the rest of components resulted in ashes and non-nitrogenous extract (Table 1). High amount of protein can be explained by the acid-alkali pretreatment used, which are usually carried out to reduce the collagen loss by excluding the effect of endogenous collagen proteases [21,26]. The protein content significantly increased (*p* < 0.05) from 86.9 to 99.4% (dry basis) in SGL and SGH as the processing pH increased from 3 to 5, respectively. Conversely, the non-nitrogenous extract decreased from 12.3 to 0.0% (dry basis) when the pH was increased, in a similar way as reported previously in a work where the effect of pH and the extraction time on structural features of SG were studied [5]. Differences in protein content could be explained by the use of excess acid during the extraction which over-hydrolyses collagen molecules causing the loss of recoverable protein [27,28]. Moreover, the non-nitrogenous fraction could include aldehydes and other carbonyl compounds obtained by deamination of free amino acids and gelatin peptides generated during the collagen hydrolysis conducted under excess of acid [29].

On the other hand, the ash content has been related with mineral content (e.g., calcium and phosphate) in the fish scales which are easily dissolved under acidic conditions [30]. Ash content could also reflect the amount of salts added for pH adjustment during extraction (e.g., NaOH). Thus, the low ash content of our SG samples would be related with salts added for pH control during processing, rather than salts brought by fish scales. Interestingly fat was not detected, indicating fat content was below the reported detection limit of the technique (~0.52 g/100 g). The alkaline pretreatments steps of the skins may have also removed the fat present.

### 3.2. Molecular Weight

Average MW of the SG polymer chains was determined by capillary viscometry [12]. SGH presented an average MW of 172.7 kDa, whereas SGL showed MW value of 64.6 kDa (Table 2). Quero et al. [12] reported a molecular weight of 85.6 kDa for gelatin produced at pH 4, which is fairly similar to the value 94.5 kDa reported in this study for SGM. Interestingly, this method showed to be sensitive to the rupture of the elemental polymer chains of SG during the extraction process, which was especially clear at low pH (SGL).

**Figure 1:**
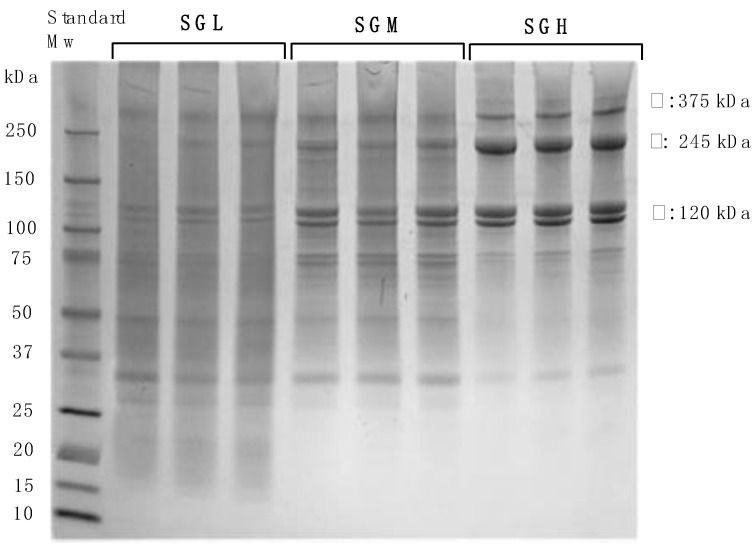
SDS-PAGE electrophoresis of SG samples; SGL, SGM, and SGH with controlled molecular weight.

The MW distribution of the gelatins determined by SDS-PAGE electrophoresis are shown in Figure 1. As expected, the degree of hydrolysis of the SG polymer chains during the extraction was strongly affected by the pH, where more acidic conditions produced polymer chains with a wider MW distribution. SDS-PAGE gel resulted in SGH presenting well-defined molecular weight bands located in the range from 100 kDa to 250 kDa, which is most likely related to α-chains (~125 kDa) and β-chains (~250 kDa). It has been proposed that covalent crosslinks between the α-chains in the native collagen fibrils can withstand the extraction condition used, resulting in fractions of β-chains (two covalently crosslinked α-chains) and γ-chains (three covalently crosslinked α-chains, ~375 kDa) [31]. On the other hand, SGL clearly showed the absence of bands associated with high MW (~250 kDa). In fact, most of the bands attributed to SGL were identified in the range between ~15 kDa and ~100 kDa (Figure 1). The absence of bands at higher MW, demonstrates the hydrolytic effect of excess acid during gelatin extraction. In the case of SGM, the bands show an intermediate and broader weight distribution, which was reported previously by Díaz-Calderón et al. [5]. An average MW estimated from the central point of MW distribution shows good agreement with the MW value reported previously by capillary viscometry.

### 3.3. Amino Acidic Profile 

The amino acidic compositions of SGL, SGM, and SGH with controlled molecular weight are presented in Table 3. The predominant amino acids found were glycine, glutamic acid, alanine, arginine, and the imino acids proline and hydroxyproline (Table 3). Interestingly, the concentration of glycine, proline, and hydroxyproline resulted higher in SGH, in agreement with higher protein content observed in SGH. These results are consistent with the work of Weng et al. [3] who observed an increase in hydroxyproline content at more alkaline pH processing (7 and 9). Presumably the over-hydrolysis resulted by excess in acid extraction and post deamination of free amino acids and gelatin peptides could explain this result. Similar amino acid composition was reported for salmon gelatin hydrolysates of low molecular weight (<10 kDa) processed using an extraction condition of 50 °C and pH 7 [32].

The cold water fish gelatin normally have lower hydroxyproline content than warm water fish and mammal gelatins (e.g., 9.6 g/100 g_protein_ in seabass [2], 10–12 g/100 g_protein_ in bovine [14,33], respectively), and resulting in low gelling temperatures. This difference could be related to the physiological adaptation of fish to their environmental temperature at which stable collagen may be required for the survival of fish, which finally plays a key role in how collagen is structuring under these conditions [33,34].

Hydroxyproline is involved in interchain hydrogen bonding, promoting and stabilizing the triple helix formation in polymer structures like-collagen [2,35]. Therefore, the content of hydroxyproline has been related to the amount of helical structures and the kinetic of triple helix formation, which may affect the structural features of SG and thermal and rheological behavior of the material [13].

### 3.4. Isoelectric Point

Although the procedure to obtain the SG samples used in this study had a significant effect on molecular weight and amino acid profile, this was not reflected in the isoelectric point (pI) of SG, which showed no significant differences among the gelatins produced (Table 2, Figure S1). The pI values ranged between 9.6 and 9.8, which was expected based on the processing pH used. Normally, the pI values are around 9 for gelatins type-A or produced under acidic conditions, and close to 5 for gelatin type-B, which are obtained through alkaline processes. The use of alkaline or acidic extraction conditions depends on the raw material to be processed, with the acidic treatment being most suitable for the less covalently crosslinked collagens like those found in fish skins [35]. The latter would explain the similar pI values reported by our study as all the gelatins were obtained at pH values equal or below pH 5. Suspensions of these SG samples prepared at pH values below the pI, the polymer chains would be positive electrically charged, hence enhancing hydration and solubility [36].

### 3.5. Molecular Configuration

**Figure 2:**
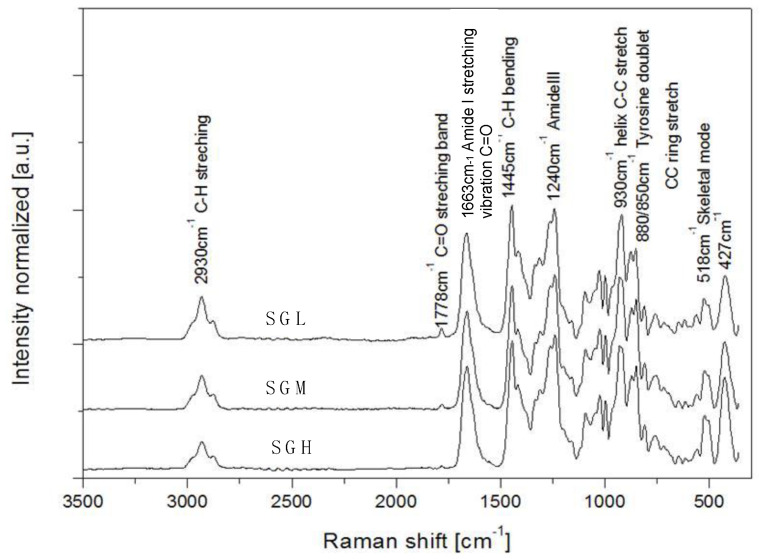
Raman spectroscopy of SG samples with controlled molecular weight.

All SG samples were evaluated in terms of their molecular structure and characteristic chemical vibrations (Figure 2). As expected, the Raman spectra showed well-defined peaks at the Amide regions, yielding four sharp peaks at 1663 cm^−1^ (Amide-I, stretching vibration of C=O), 1445 cm^−1^ (C-H bending), 1240 cm^−1^ (Amide-III, associated with coupled C-N stretching and N-H bending vibrations), and 930 cm^−1^ (helix stretching C-C) [37]. The peaks associated with structures type Amide I and III are characteristic of secondary structure of proteins and their presence indicate the helical configuration of the SG adopted under different molecular weight conditions [38,39,40]. The presence of these peaks in all the gelatins is consistent with the fact that gelatin films samples were prepared by cold casting method which promote the formation of helical structures upon cooling.

The main differences among the SG samples in the Raman spectra were found at 2900 cm^−1^, 1778 cm^−1^, 518 cm^−1^, and 427 cm^−1^. The peak at 2900 cm^−1^ corresponds to the C-H stretching vibration and SGL showed greater intensity compared to SGH (Figure 2). In protein-based systems, increases in the intensity of C-H stretching vibrations at extreme pH (e.g., pH 3) are indicative of protein denaturation [40]. Thus, the intensity can be attributed to the unfolding of the SG polymer chain which promotes a higher exposure of hydrophobic groups to a more polar environment at more acidic pH conditions. Hence, differences in intensity are suggesting that GS with higher molecular weight, protein, and imino acids content (e.g., SGH) are organized under more complex helical structures. Additionally, the higher Raman band intensity at 518 cm^−1^, 530 cm^−1^, and 427 cm^−1^ can be related to C-O-C and C-C-O skeletal vibration, which could be attributed to changes in hydrophobic interactions in the backbone, specifically to aliphatic and tryptophan aromatic residues less exposed to the solvent as has been reported in the literature [41,42].

The peak located at 1778 cm^−1^ would be not related to any protein chemical structure vibration and according to the literature it could be associated to the presence of acetic acid in the more hydrolyzed gelatin samples [42] and shown by SGL and SGM.

### 3.6. Thermal Properties

The gelling temperature (°C) and gelation enthalpy (ΔH) of the SGL, SGM, and SGH samples are presented in Table 2. The results showed that gelling temperature decreased from 10.3 °C to 3.3 °C when MW changed from ~173 kDa to ~65 kDa, respectively. Moreover, the higher MW of SGH showed higher gelation enthalpy, which is expected, whereas more hydrolyzed gelatins (SGM and SGL) show a lower gelation enthalpy. Indeed, SGL presented a gelation enthalpy value of −1.8 J g^−1^, while SGH showed −5.8 J g^−1^ for this transition. Correlations between gelling temperature, gelation enthalpy, and processing conditions have been previously reported in the literature, and have been explained in terms of the triple helix content in the gelatin which is strongly dependent on the MW distribution of the polymer, but also because the lower junction zones or entanglement of molecule is lower for gelation with low MW chain than the high MW counterpart [5,43,44,45].

The literature has widely correlated gelatin MW with gelation enthalpy and gel strength features. Gel strength has been usually related with the ability of the gelatin to form sufficient helical structures for network formation and stability. Indeed, a linear correlation has been reported between gel strength and the amount of triple-helices present in gelatin-based materials [35,46]. In our study the highly hydrolyzed SGL sample showed very low gel strength whereas SGH formed a stronger gel (Figure S2). In addition, MW of the SG samples showed a direct correlation with the gel strength and gelation enthalpy. Therefore, these results would indicate that lower MW chains could no longer contribute to form stable gels because of the reduction in the number of helical and entangled structures present in the system. Supporting this hypothesis, a recent study by Díaz-Calderón et al. [5] looking at salmon gelatin extracted at various pH values and processing times, described a reduction in the peak intensity at ~8° observed by wide angle X-ray diffraction pattern for the lowest MW sample tested, which was associated to the formation and stacking of helical structures in the gelatin. In this study, differences in molecular configuration in the gelatin samples were also reflected by the Raman spectra (C-H stretching vibrations, Figure 2), which being consistent with the proximate composition and imino acids content (Table 1 and Table 2), would support the idea that SGL consists in less complex organization in terms of helical structure formation.

### 3.7. Rheological Behavior

#### 3.7.1. Steady-Shear Flow Measurements

**Figure 3:**
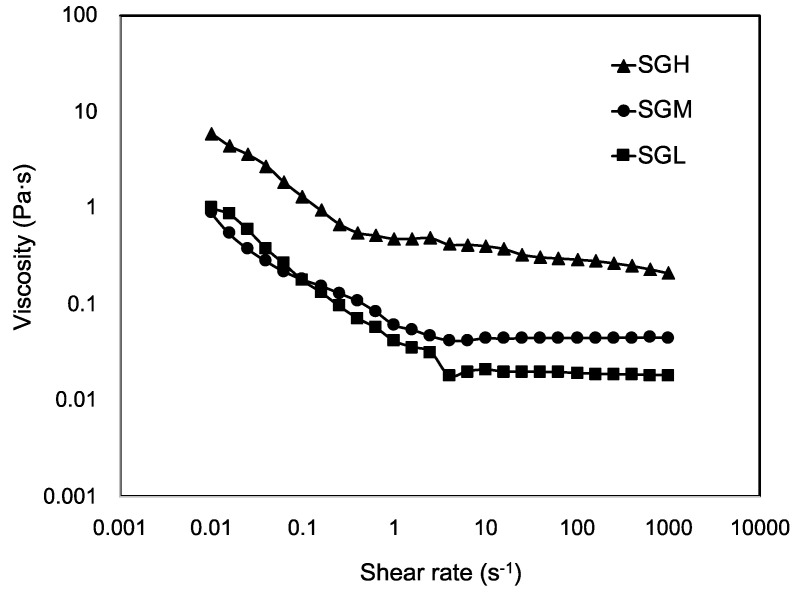
Steady shear viscosity (25 °C) of SG samples with controlled molecular weight.

Steady shear viscosity values for SGL, SGM, and SGH samples (measured at 25 °C) are shown in Figure 3. A significant difference in viscosity was observed at high values of shear rate (>10 s^−1^), showing higher viscosity values for SGH. All samples showed two well-defined zones of viscosity-shear rate dependence, with SGL and SGM showing similar behavior. At increasing shear rates from 0.01 s^−1^ to 3 s^−1^, both SGL and SGM showed a shear thinning behavior with a continuous decrease of viscosity. This behavior has been explained by a continuous alignment of the highly anisotropic chains in the direction of the shear rate [46]. The second viscosity zone for shear rates values higher than 4 s^−1^, showed both gelatins reaching a Newtonian plateau, which suggest a dimensional stability related with SG chains forming a firm structure as the viscosity curve approaches infinity on a constant slope [47]. In the case of SGM the viscosity was significantly higher than SGL, being ~0.045 Pa·s and ~0.019 Pa·s, respectively. In the case of SGH, a similar shear thinning effect was observed but reporting higher viscosity values. Interestingly, the shear rate value at which the viscosity dependence changed to Newtonian behavior was lower (0.4 s^−1^), which would be explained by the higher MW and smaller MW distribution in SGH, increasing the probability of chains to join each other, hence promoting the gelatin stabilization by the organization in a firm structure at lower values of shear rate, in a behavior that can also be influenced by the higher amount of imino acids present in SGH.

Similar findings has been reported by Huang et al. [48] for gelatin from carp scales using an extraction protocol assisted by an ultrasonic stage. Authors reported a shear thinning and Newtonian behavior with viscosity values around 0.02–0.08 Pa·s at the Newtonian zone. This result was associated with changes in gels dispersity, molecular shape, and strength of the intra-molecular chemical bonds upon various shear rates [48,49].

#### 3.7.2. Temperature Sweep Tests

**Figure 4:**
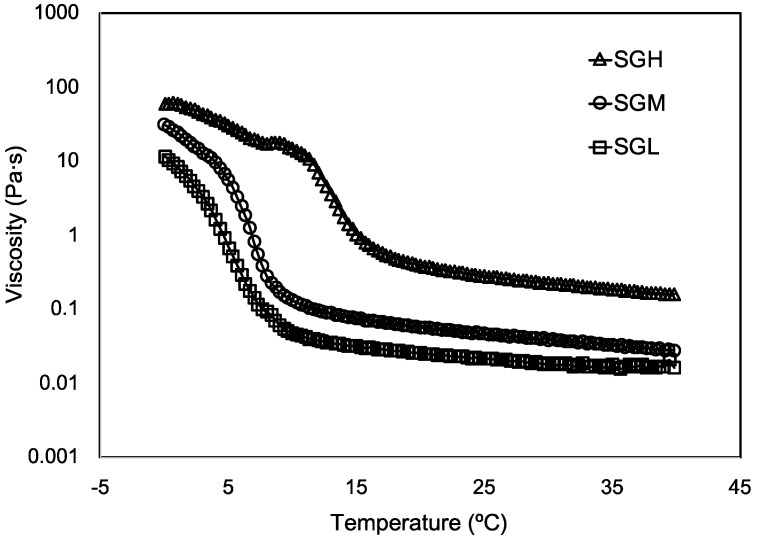
Flow temperature sweep of SG samples with controlled molecular weight.

The effect of the controlled molecular weight on the viscosity of the SG samples was evaluated as a function of temperature upon cooling (Figure 4). As expected, the apparent viscosity of all the gelatins showed a strong dependence on temperature. In fact, a significant increase of about three orders of magnitude in viscosity was observed at temperature around 15 °C in SGH but around 8 °C in SGM and SGL. This increase in viscosity could be explained by the system reaching a temperature below the gelling point at which the well-known conformational change associated to the coil-to-helix transition and the formation of a network featuring molecular ordered elements occur. The latter would be mainly driven by physical interaction intra and inter α-chains leading to the formation of triple helices structures [50,51]. These changes in viscosity at different temperatures in the three gelatins samples correlated with the reported values by DSC in Table 2 in terms of controlled gelling temperatures with molecular weight.

SGL presented a lower viscosity than SGH. Indeed, at 25 °C viscosities were 0.021 and 0.281 Pa·s, respectively (Table 2). Differences in viscosity values among the SG samples were also observed at lower temperatures. At 4 °C the viscosity values were 1.6 Pa·s, 9.7 Pa·s, and 36.2 Pa·s for SGL, SGM, and SGH, respectively (Table 2).

The differences in viscosity at the temperature range tested between the gelatins could also be explained by the observed differences in MW distribution, average MW, and possible number of triple helical structures, as has been extensively reported in the literature [1,13,31,44,52]. Sperling [52] stated the idea that viscosity can be partially controlled by the average MW and molecular size distribution of the protein. Our study supports this behavior, since a direct correlation was found between both MW and MW distribution of SG; the viscosity values are reported in Figure 3 and Figure 4. Even in our study this behavior seems to be also influenced by the amino acid composition. This mechanical behavior is interesting since low viscosity and low gelling temperature SG could provide a technological advantage over mammal gelatins at high concentration since it retains a suitable viscosity for applications relevant to foods, pharma, and biomedicine among other application [8,10,53]. For example, a novel sprayable photo-sensitive SG-based coating has been recently described by Char et al. [4], where the low viscosity and low gelling temperature of SG allowed the application of this coating under low temperature conditions normally used by the food industry (T < 6 °C), and therefore easing the adoption of this technology by the industry. On the other hand, Zaupa et al. [10] have reported the technical advantages of SG for use with biofabrication purposes over mammal gelatin, where a combination of low gelling temperature, higher molecular mobility (relaxation times T2 by NMR), and lower capacity of SG to thermal inducing random coil to triple helix transformation upon cooling (circular dicroism), resulted in important advantages for tuning the rate of extra cellular matrix remodeling, which is relevant for wound healing treatments and tissue engineering applications. Hence, the biofabrication of constructs under different 3D printing configurations (e.g., layer-by-layer and ink polyjet deposition) could be abled by the use of low viscosity gelatins such as SG.

**Figure 5:**
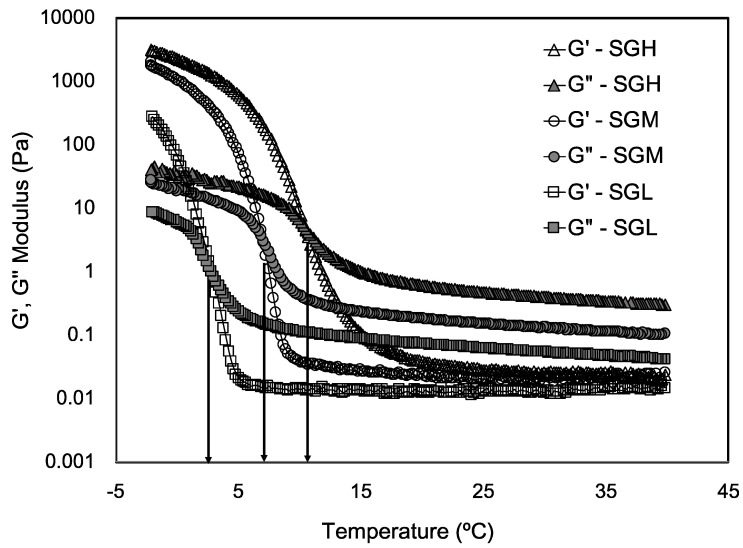
Viscoelastic parameters (G′ and G′′) of salmon gelatin with controlled molecular weight. Both moduli were assessed upon cooling and arrows indicate the gelling temperature of each SG sample.

Temperature sweeps under oscillatory conditions at constant strain reporting the storage and loss moduli (G′ and G′′, respectively) of SGL, SGM, and SGH are depicted in Figure 5. The sol-gel transition temperature was clearly identified as the intersection point between the G′ and G” curves, [46,54]. The gelling temperatures obtained were in average 2.8, 7.0, and 10.3 °C for the SGL, SGM, and SGH, respectively, which are in agreement to those reported by DSC (Table 2). From Figure 5 we can see that for all the gelatins at temperatures higher than the gelling point, G” values were higher than G′ values, indicating a significant contribution of the viscous component and therefore all gelatin systems behaved mechanically as a liquid. Whereas the opposite was observed at temperatures below the gelling point (G′ > G′′), where the sol-gel transition already occurred generating a solid-like SG system. An interesting feature in these curves is the difference between G′ and G′′ values after the sol-gel transition is reached at the lowest temperature tested (~0 °C). This difference becomes smaller for SGL compared to SGH. Indeed, the difference between both moduli was 39.17 Pa (44.2–5.03 Pa) for SGL and 1987.9 Pa (2018.8–30.9 Pa) in the case of SGH. Although this behavior is influenced by the kinetic of self-association of gelatin strands at low temperature, this result could be also related to more complex structures present in the gelatin with higher molecular weight and with higher contribution to the elastic component in gelatin-based gels. A similar behavior was observed in the liquid-like zone (G′′ > G′), which was consistent with the higher viscosity of SGH previously reported in this work (Figure 3 and Figure 4). These results show a direct correlation with data previously observed in terms of MW distribution, proximate composition, molecular configuration, and gel strength (Figure 1 and Figure 2, Table 2). Therefore SGH, which featured higher MW, showed higher G′ and structured molecular configuration.

#### 3.7.3. Frequency Sweep Test

**Figure 6:**
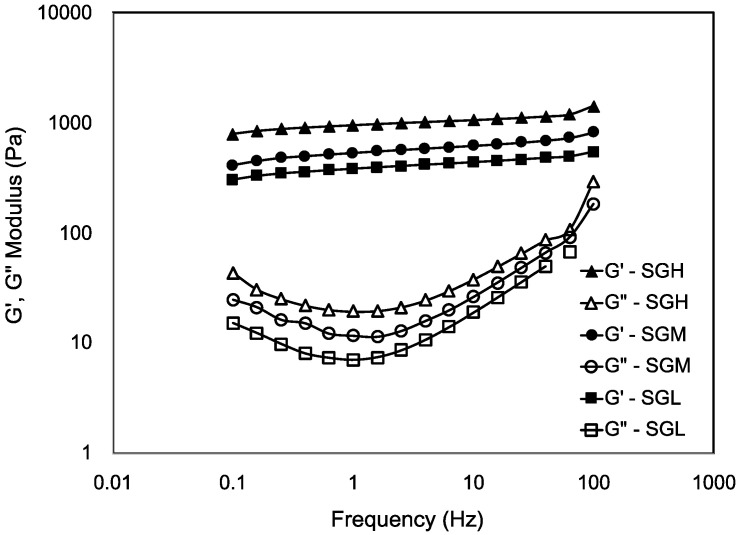
Oscillatory frequency sweep of salmon gelatin with controlled molecular weight. All samples tested 5 °C below the gelling temperature assessed by rheology.

The viscoelasticity of the gels of SGL, SGM, and SGH by oscillatory frequency sweeps assessed at 5 °C below the gelling temperature are presented in Figure 6. All SG samples showed the elastic modulus (G′) being significantly higher than the loss modulus (G”) throughout all the frequency range tested (0.1–100 Hz). G′ values showed a subtle increase when the frequency was increased, whereas the G′′ values showed strong frequency dependence. According to Ikeda and Nishinari [54], a frequency dependence on the moduli and values of tan δ > 0.1 could represent the so-called weak gels. However, our results show that independently of the MW and molecular configuration (helical structures) of the produced SG samples, they all behave as strong gels in terms of their viscoelasticity (tan δ < 0.1, Figure S3). Frequency sweep in all SG gels resulted in G′ values of about two order of magnitude higher than G′′ values, indicating a structured gel network with a solid-like mechanical response to deformation [48] (Figure 6 and Figure S3, respectively). For example, tan δ values obtained at 1 Hz were similar among the gelatin samples with values of 0.018, 0.022, and 0.021 for SGL, SGM, and SGH, respectively (data not shown). However, tan δ resulted in higher values at higher frequencies (Figure S3) indicating that SG trends toward to organize in weak gel network at short deformation time [55]. At low frequencies and therefore at longer times, SG gel behaves as a strong gel. Similar findings has been reported in other gelatin-based systems [49,56,57,58].

Figure 6 also show higher moduli (G′ and G”) in the higher molecular weight gelatins. These results support the fact that higher MW and higher amount of helical structures (α- and β-chains) in the gelatin promote a more rigid structure, which is consistent with the reported gelation enthalpy and gel strength. Chandra and Shamasundar [56] have explained the condition of strong gel network of gelatin-based system by higher stability reached by gelatin strands at the junction zones after the gelation process. The literature has also suggested that differences in viscoelastic properties could not be exclusively explained by differences in amino acid content [53,58,59]. Our results support that hypothesis, as the concentration of imino acids proline and hydroxyproline in the SGL and SGH showed statistical differences (Table 3), but also the degree of hydrolysis generated during the gelatin production affected the proximate composition, the average molecular weight, the molecular weight distribution and the molecular configuration of gelatin strands, therefore, these factors appear to be also as important factors to affect the rheological properties of SG samples, including viscosity, G′ and G” values. In addition, the presence of different hydrolytic fragments in fish gelatin have also been reported to influence the G′ and gel strength [60].

The fact that GS behaves as strong gels in terms of viscoelasticity for the MW and MW distribution tested, strongly supports and encourages the potential uses of SG in biomedical applications. Specifically, in terms of tissue engineering the performance of a GS-based construct developed by 3D printing or analogous technologies, could be complementarily explained by the viscoelastic response of gelatin. Thus, despite of the lower stiffness showed by SG over mammal gelatins reported by Zaupa et al. [10] and its low viscosity, SG gels behave as strong gel which promotes the different physiological processes of cell, therefore improving the mechanisms involved in integration and regeneration of polymer scaffolds. Interestingly, this effect showing to be independent of the MW of SG highlights the advantages of SG as a biomaterial for the design of applications relevant to electrospinning, microfluidics, and micropatterning which currently require further development (e.g., 3D printing). Additionally, some of these applications consider the use of different stabilization strategies of the polymer structures to enhance the mechanical response of the construct (e.g., photo-crosslinking). Thus, using strong viscoelastic hydrogels based on SG in combination with different mechanism of stabilization could provide a way to tailor the mechanical resistance and get controlled degradability of polymer construct based on this novel biomaterial.

## 4. Conclusions

This study showed the direct correlation existing between MW and molecular structure of SG with macromolecular properties such as the gelation energy and rheological performance. The high molecular weight of salmon gelatin (SGH) was directly correlated with the highest viscosity, gelling temperature, and G′. Raman spectra and gelation energy suggest a higher amount of helical structures in this SG sample. Conversely, SGL showed lower viscosity and G′, most possibly because of the hydrolytical conditions addressed during sample preparation allowed the rupture of elemental chains resulting in low MW chains with less capability to organize forming helical structures.

Interestingly, despite the low gelling temperature and low viscosity which allow to SG to flow at room temperature, it behaved as a strong gel in terms of viscoelasticity once it is structured in hydrogels. Although our study showed clear differences in G′ among SG samples assessed by the frequency sweep test, the strong gel condition resulted to be independent of MW tested and molecular configuration because it was observed in all three gelatin samples tested, even in SGL.

Therefore, our study highlights the role of MW and molecular configuration for structuring gelatin-based matrices and thereafter with the performance of this material at the macroscale. We believed this analytical approach is valuable in providing further information on the structure-function relationship, especially on GS and potentially on other gelatins from cold-adapted fish. Hence, our study suggests the feasibility in defining the thermal and rheological properties of SG throughout tuning by MW through extraction processing conditions.

These results open interesting perspectives for the design of low viscosity gelatin-based materials with improved properties to be required for applications in any high-value applications that requires fine structuring using low viscosity materials such as electrospinning, microfluidics, micropatterning, and ink-jet bioprinting.

## Figures and Tables

**Table 1:** Proximate composition of salmon gelatin samples used in this study. SGX, X = molecular weight defined by pH used during extraction process. Values in brackets correspond to standard deviation (n = 3).

Component	SGL	SGM	SGH
**Moisture (%, wb) ^1^**	6.6 (0.0) ^a^	4.0 (1.5) ^b^	3.9 (0.2) ^c^
**Protein (%, db) ^2^**	86.9 (4.2) ^a^	93.9 (0.4) ^b^	99.4 (0.07) ^c^
**Non-nitrogenous Extract (%, db) ^3^**	12.3 (4.1) ^a^	5.5 (0.4) ^b^	0.0 (0.0) ^c^
**Fat (%, db)**	ND ^4^	ND	ND
**Ash (%, db)**	0.8 (0.08) ^a^	0.6 (0.01) ^b^	0.6 (0.07) ^b^

^a,b^ Within the same row, different superscript lowercase letters show significant difference (*p* < 0.05). ^1^ wb, wet basis; ^2^ db, dry basis; ^3^ %N × 5.5; ^4^ ND, non-detected by analysis.

**Table 2:** Physicochemical properties of SG samples; low (SGL), medium (SGM) and high (SGH) controlled molecular weight (MW, by capillary viscometry): isoelectric point (pI), gelling temperature by calorimetry, and rheology, gelation enthalpy, and viscosity (at 4 °C and 25 °C). Values in brackets correspond to standard deviation (n = 3). Within the same column, different superscript lowercase letters show significant difference (*p* < 0.05).

Sample	MW (kDa)	pI	T_gelling_ (°C, by DSC)	ΔH (J g^−1^_dry sample_)	Viscosity (Pa·s)		T_gelling_ (°C, by Rheology)
					4 °C	25 °C	
**SGL**	64.6 (14) ^a^	9.7 (0.5) ^a^	3.3 (0.5) ^a^	−1.8 (0.7) ^a^	1.6 (1.2) ^a^	0.02 (0.003) ^a^	2.8 (0.01) ^a^
**SGM**	94.5 (2.5) ^b^	9.6 (0.7) ^a^	6.9 (0.8) ^b^	−4.0 (0.7) ^b^	9.7 (0.1) ^b^	0.05 (0.001) ^b^	7.0 (0.41) ^b^
**SGH**	172.7 (26) ^c^	9.8 (0.1) ^a^	10.3 (0.6) ^c^	−5.8 (2.1) ^b^	36.2 (4.5) ^c^	0.28 (0.013) ^c^	10.3 (0.73) ^c^

**Table 3:** Amino acidic profile (g/100 g_protein_) of SG with controlled molecular weight. Values in brackets correspond to standard deviation (n = 3). Within the same row, different superscript lowercase letters show significant difference (*p* < 0.05).

	g/100 g_protein_		
Amino Acid	SGL	SGM	SGH
alanine	6.04 (0.6) ^a^	6.27 (0.1) ^a^	6.02 (1.4) ^a^
arginine	5.36 (0.7) ^a^	6.13 (0.4) ^b^	7.06 (0.2) ^c^
aspartic acid	4.35 (0.3) ^a^	5.08 (0.2) ^b^	3.98 (0.3) ^a^
glutamic acid	8.13 (1.1) ^a^	8.15 (0.0) ^a^	9.61 (0.0) ^b^
glycine	18.88 (3.2) ^a^	18.54 (0.3) ^a^	25.23 (2.1) ^b^
hydroxiproline	5.87 (0.8) ^a^	5.94 (0.1) ^a^	8.06 (1.1) ^b^
hysitidine	0.33 (0.1) ^a^	0.59 (0.1) ^a^	0.26 (0.4) ^a^
isoleucine	0.65 (0.0) ^a^	0.73 (0.0) ^b^	0.52 (0.3) ^a,b^
leucine	1.33 (0.0) ^a^	1.47 (0.0) ^b^	1.03 (0.6) ^a,b^
lysine	2.94 (0.4) ^a^	3.01 (0.0) ^a^	2.87 (0.7) ^a^
methionine	1.25 (0.1) ^a^	1.38 (0.0) ^a^	1.24 (0.5) ^a^
phenylalanine	1.18 (0.0) ^a^	1.28 (0.0) ^b^	1.31 (0.0) ^b^
proline	6.76 (1.2) ^a^	6.93 (0.2) ^a^	10.57 (1.4) ^b^
serine	3.05 (0.4) ^a^	3.14 (0.0) ^a^	4.00 (0.3) ^a^
treonine	1.33 (0.1) ^a^	1.44 (0.0) ^a^	1.52 (0.1) ^a^
valine	0.61 (0.1) ^a^	0.94 (0.2) ^a^	0.47 (0.7) ^a^

## Supplementary Materials

The following are available online at https://www.mdpi.com/2073-4360/12/7/1587/s1, Figure S1: Isoelectric point of salmon gelatin with controlled molecular weight, Figure S2: Gel strength of salmon gelatin with controlled molecular weight, Figure S3: Viscoelastic parameter Tan δ of salmon gelatin with controlled molecular weight tested by frequency sweep.

## References

[1] Lin L., Regenstein J.M., Lv S., Lu J., Jiang S. (2017). An overview of gelatin derived from aquatic animals: Properties and modification. Trends Food Sci. Technol..

[2] Sinthusamran S., Benjakul S., Kishimura H. (2015). Molecular characteristics and properties of gelatin from skin of seabass with different sizes. Int. J. Biol. Macromol..

[3] Weng W., Zheng H., Su W. (2014). Characterization of edible films based on tilapia (Tilapia zillii) scale gelatin with different extraction pH. Food Hydrocoll..

[4] Char C., Padilla C., Campos V., Pepczynska M., Paulo D., Enrione J. (2019). Characterization and testing of a novel sprayable crosslinked edible coating based on salmon gelatin. Coatings.

[5] Díaz-Calderón P., Flores E., González-Muñoz A., Pepczynska M., Quero F., Enrione J. (2017). Influence of extraction variables on the structure and physical properties of salmon gelatin. Food Hydrocoll..

[6] Gullapalli R.P., Mazzitelli C.L. (2017). Gelatin and non-gelatin capsule dosage forms. J. Pharm. Sci..

[7] Pal A., Bajpai J., Bajpai A.K. (2018). Poly (acrylic acid) grafted gelatin nanocarriers as swelling controlled drug delivery system for optimized release of paclitaxel from modified gelatin. J. Drug Deliv. Sci. Technol..

[8] Enrione J., Pino K., Pepczynska M., Brown D.I., Ortiz R., Sánchez E., Acevedo C.A. (2018). A novel biomaterial based on salmon-gelatin and its in vivo evaluation as sterile wound-dressing. Mater. Lett..

[9] Enrione J., Blaker J.J., Brown D.I., Weinstein-Oppenheimer C.R., Pepczynska M., Olguín Y., Sánchez E., Acevedo C.A. (2017). Edible scaffolds based on non-mammalian biopolymers for myoblast growth. Materials.

[10] Zaupa A., Byres N., Dal C., Acevedo C.A., Angelopoulos I., Terraza C., Nestle N., Abarzúa-illanes P.N., Quero F., Díaz-Calderón P. (2019). Cold-adaptation of a methacrylamide gelatin towards the expansion of the biomaterial toolbox for specialized functionalities in tissue engineering. Mater. Sci. Eng. C.

[11] Chiou B.S., Avena-Bustillos R.J., Bechtel P.J., Imam S.H., Glenn G.M., Orts W.J. (2009). Effects of drying temperature on barrier and mechanical properties of cold-water fish gelatin films. J. Food Eng..

[12] Quero F., Padilla C., Campos V., Luengo J., Caballero L., Melo F., Li Q., Eichhorn S.J., Enrione J. (2018). Stress transfer and matrix-cohesive fracture mechanism in microfibrillated cellulose-gelatin nanocomposite films. Carbohydr. Polym..

[13] Joly-Duhamel C., Hellio D., Djabourov M. (2002). All gelatin networks: 1. Biodiversity and physical chemistry. Langmuir.

[14] Díaz P., López D., Matiacevich S., Osorio F., Enrione J. (2011). State diagram of salmon (Salmo salar) gelatin films. J. Sci. Food Agric..

[15] Kwak H.W., Shin M., Lee J.Y., Yun H., Song D.W., Yang Y., Shin B.S., Park Y.H., Lee K.H. (2017). Fabrication of an ultrafine fish gelatin nanofibrous web from an aqueous solution by electrospinning. Int. J. Biol. Macromol..

[16] Acevedo C.A., Orellana N., Avarias K., Ortiz R., Benavente D., Prieto P. (2018). Micropatterning technology to design an edible film for in vitro meat production. Food Bioprocess. Technol..

[17] Orellana N., Elizabeth S., Benavente D., Prieto P., Enrione J., Acevedo C.A. (2020). A new edible film to produce in vitro meat. Foods.

[18] Abdelhedi O., Jridi M., Nasri R., Mora L., Toldrá F., Nasri M. (2019). Rheological and structural properties of Hemiramphus far skin gelatin: Potential use as an active fish coating agent. Food Hydrocoll..

[19] Billiet T., Gevaert E., De Schryver T., Cornelissen M., Dubruel P. (2014). The 3D printing of gelatin methacrylamide cell-laden tissue-engineered constructs with high cell viability. Biomaterials.

[20] Otoni C.G., Avena-Bustillos R.J., Chiou B.S., Bilbao-Sainz C., Bechtel P.J., McHugh T.H. (2012). Ultraviolet-B radiation induced cross-linking improves physical properties of cold- and warm-water fish gelatin gels and films. J. Food Sci..

[21] Hou P.Z., Regenstein J.M. (2006). Optimization of extraction conditions for pollock skin gelatin. J. Food Sci..

[22] Association of the Official Analytical Chemists (AOAC) (2016). Official Methods of Analysis of AOAC International.

[23] Harding S.E. (1997). The intrinsic viscosity of biological macromolecules. Progress in measurement, interpretation and application to structure in dilute solution. Prog. Biophys. Mol. Biol..

[24] Enrione J.I., Sáez C., López D., Skurtys O., Acevedo C., Osorio F., MacNaughtan W., Hill S. (2012). Structural relaxation of salmon gelatin films in the Glassy State. Food Bioprocess. Technol..

[25] Rebane R., Herodes K. (2010). A sensitive method for free amino acids analysis by liquid chromatography with ultraviolet and mass spectrometric detection using precolumn derivatization with diethyl ethoxymethylenemalonate: Application to the honey analysis. Anal. Chim. Acta.

[26] Neves A.C., Harnedy P.A., O’Keeffe M.B., Alashi M.A., Aluko R.E., FitzGerald R.J. (2017). Peptide identification in a salmon gelatin hydrolysate with antihypertensive, dipeptidyl peptidase IV inhibitory and antioxidant activities. Food Res. Int..

[27] Jamilah B., Harvinder K.G. (2002). Properties of gelatins from skins of fish-black tilapia (Oreochromis mossambicus) and red tilapia (Oreochromis nilotica). Food Chem..

[28] Niu L., Zhou X., Yuan C., Bai Y., Lai K., Yang F., Huang Y. (2013). Characterization of tilapia (Oreochromis niloticus) skin gelatin extracted with alkaline and different acid pretreatments. Food Hydrocoll..

[29] Voet D., Voet J. (2011). Biochemistry.

[30] Sankar S., Sekar S., Mohan R., Rani S., Sundaraseelan J., Sastry T.P. (2008). Preparation and partial characterization of collagen sheet from fish (Lates calcarifer) scales. Int. J. Biol. Macromol..

[31] Eysturskard J., Haug I.J., Elharfaoui N., Djabourov M., Draget K.I. (2009). Structural and mechanical properties of fish gelatin as a function of extraction conditions. Food Hydrocoll..

[32] Harnedy P.A., Parthsarathy V., McLaughlin C.M., O’Keeffe M.B., Allsopp P.J., McSorley E.M., O’Harte F.P.M., FitzGerald R.J. (2018). Atlantic salmon (Salmo salar) co-product-derived protein hydrolysates: A source of antidiabetic peptides. Food Res. Int..

[33] Liu D., Nikoo M., Boran G., Zhou P., Regenstein J.M. (2015). Collagen and gelatin. Annu. Rev. Food Sci. Technol..

[34] Santos J.P., Esquerdo V.M., Moura C.M., Pinto L.A.A. (2018). Crosslinking agents effect on gelatins from carp and tilapia skins and in their biopolymeric films. Colloids Surf. A Physicochem. Eng. Asp..

[35] Karim A.A., Bhat R. (2009). Fish gelatin: Properties, challenges, and prospects as an alternative to mammalian gelatins. Food Hydrocoll..

[36] Binsi P.K., Shamasundar B.A., Dileep A.O., Badii F., Howell N.K. (2009). Rheological and functional properties of gelatin from the skin of Bigeye snapper (Priacanthus hamrur) fish: Influence of gelatin on the gel-forming ability of fish mince. Food Hydrocoll..

[37] Zhu G., Zhu X., Fan Q., Wan X. (2011). Raman spectra of amino acids and their aqueous solutions. Spectrochim. Acta-Part A Mol. Biomol. Spectrosc..

[38] Cebi N., Durak M.Z., Toker O.S., Sagdic O., Arici M. (2016). An evaluation of Fourier transforms infrared spectroscopy method for the classification and discrimination of bovine, porcine and fish gelatins. Food Chem..

[39] Duconseille A., Gaillard C., Santé-Lhoutellier V., Astruc T. (2018). Molecular and structural changes in gelatin evidenced by Raman microspectroscopy. Food Hydrocoll..

[40] Celedón A., Aguilera J.M. (2002). Applications of microprobe Raman spectroscopy in food science. Food Sci. Technol. Int..

[41] Ellepola S.W., Choi S.M., Phillips D.L., Ma C.Y. (2006). Raman spectroscopic study of rice globulin. J. Cereal Sci..

[42] Nakabayashi T., Kosugi K., Nishi N. (1999). Liquid structure of acetic acid studied by Raman spectroscopy and Ab initio molecular orbital calculations. J. Phys. Chem. A.

[43] Elharfaoui N., Djabourov M., Babel W. (2007). Molecular weight influence on gelatin gels: Structure, enthalpy and rheology. Macromol. Symp..

[44] Badii F., MacNaughtan W., Mitchell J.R., Farhat I. (2014). The effect of drying temperature on physical properties of thin gelatin films. Dry. Technol..

[45] Chandra M.V., Shamasundar B.A. (2015). Texture profile analysis and functional properties of gelatin from the skin of three species of fresh water fish. Int. J. Food Prop..

[46] Casanovas A., Hernández M.J., Martí-Bonmatí E., Dolz M. (2011). Cluster classification of dysphagia-oriented products considering flow, thixotropy and oscillatory testing. Food Hydrocoll..

[47] Mezger T.G. (2019). Applied Rheology.

[48] Huang T., Tu Z., Wang H., Zhang L. (2017). Rheological and structural properties of fish scales gelatin: Effects of conventional and ultrasound- assisted extraction. Int. J. Food Prop..

[49] Gornall J.L., Terentjev E.M. (2008). Helix–coil transition of gelatin: Helical morphology and stability. Soft Matter.

[50] Guo L., Colby R.H., Lusignan C.P., Whitesides T.H. (2003). Kinetics of triple helix formation in semidilute gelatin solutions. Macromolecules.

[51] Haug I.J., Draget K.I., Smidsrød O. (2004). Physical and rheological properties of fish gelatin compared to mammalian gelatin. Food Hydrocoll..

[52] Sperling L.H., Sperling L. (2005). Dilute solution thermodynamics, molecular weights, and sizes. Introduction to Physical Polymer Science.

[53] Boran G., Mulvaney S.J., Regenstein J.M. (2010). Rheological properties of gelatin from silver carp skin compared to commercially available gelatins from different sources. J. Food Sci..

[54] Ikeda S., Nishinari K. (2001). “Weak Gel” -type rheological properties of aqueous dispersions of nonaggregated K-carrageenan helices. J. Agric. Food Chem..

[55] Sarbon N.M., Badii F., Howell N.K. (2013). Preparation and characterisation of chicken skin gelatin as an alternative to mammalian gelatin. Food Hydrocoll..

[56] Chandra M.V., Shamasundar B.A. (2015). Rheological properties of gelatin prepared from the swim bladders of freshwater fish Catla catla. Food Hydrocoll..

[57] Clark A.H., Ross-Murphy S.B. (1987). Structural and mechanical properties of biopolymer gels. Adv. Polym. Sci..

[58] Arnesen J.A., Gildberg A. (2007). Extraction and characterisation of gelatine from Atlantic salmon (Salmo salar) skin. Bioresour. Technol..

[59] Gudmundsson M. (2002). Rheological properties of fish gelatins. J. Food Sci..

[60] Eysturskard J., Haug I.J., Ulset A.S., Draget K.I. (2009). Mechanical properties of mammalian and fish gelatins based on their weight average molecular weight and molecular weight distribution. Food Hydrocoll..

