# Supplementary material for: Rheological and Structural Study of Salmon Gelatin with Controlled Molecular Weight
Source: Polymers (Basel). 2020 Jul 17;12(7):1587. doi: 10.3390/polym12071587 (PMC7407307; doi:10.3390/polym12071587)

Supplementary material

Figure 1.SM. Isoelectric point of salmon gelatin with controlled molecular weight: a) SGL, b) SGM and c) SGH.

a) SGL

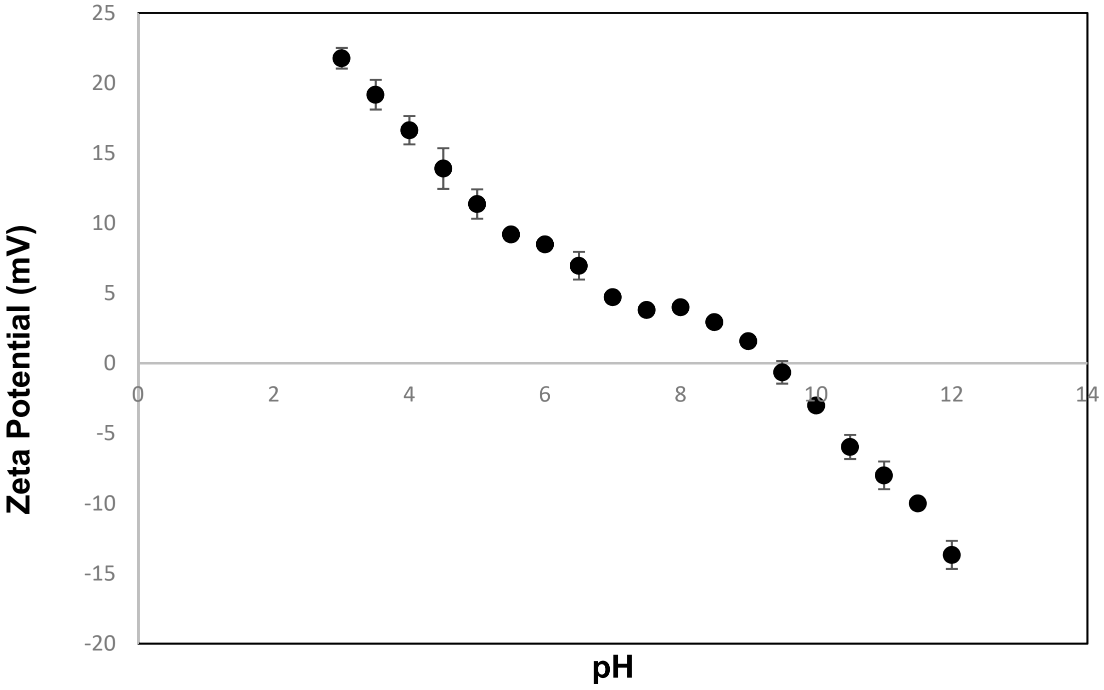

b) SGM

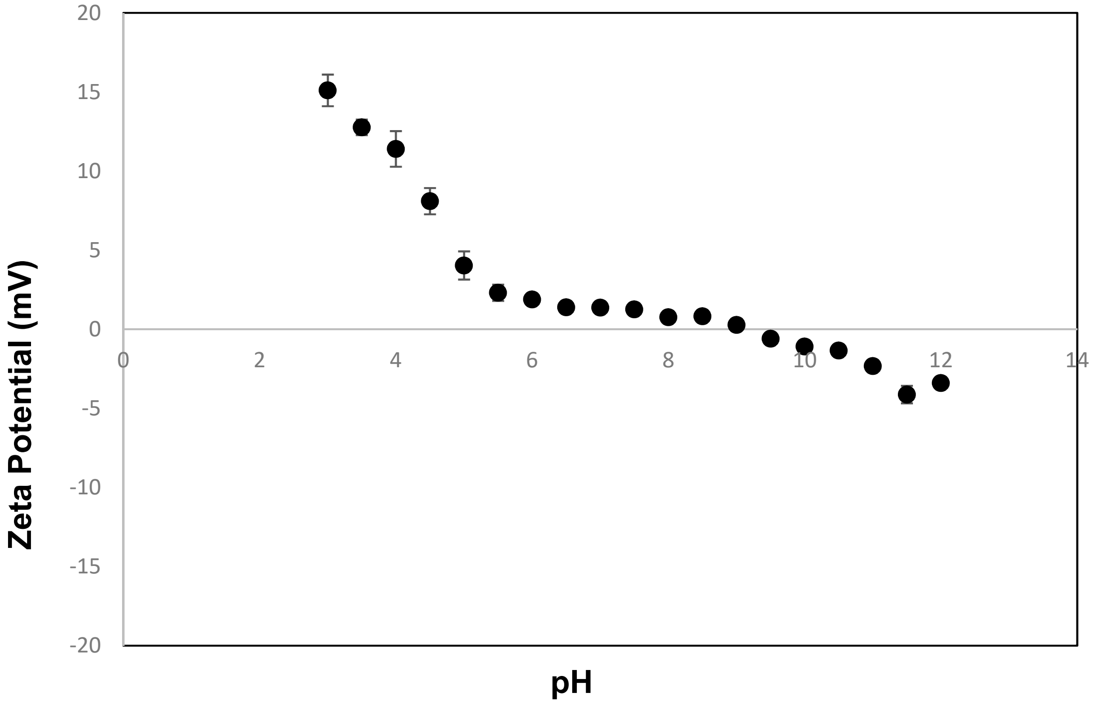

c) SGH

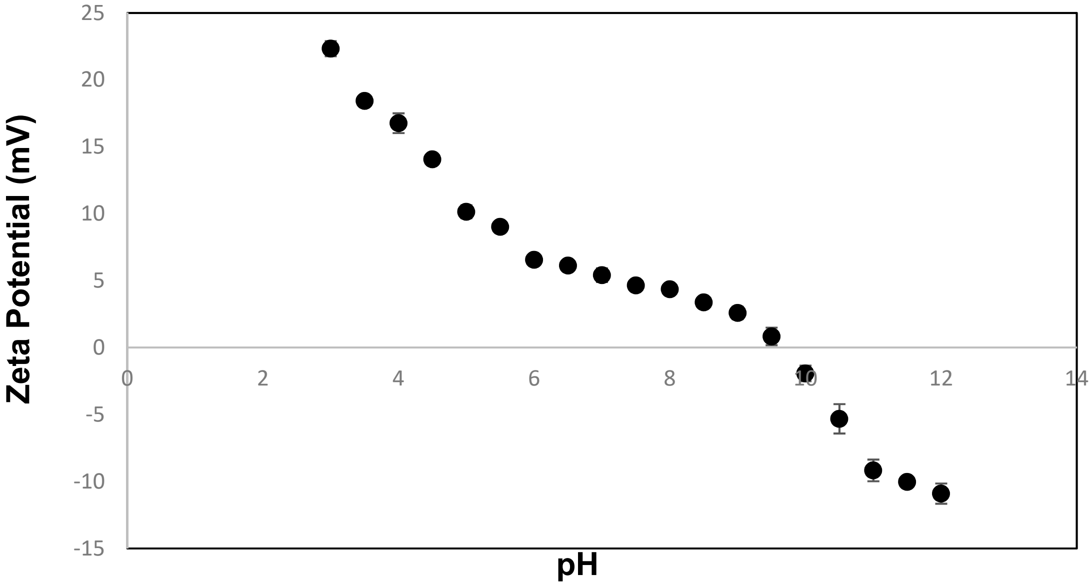

Figure 2.SM: Gel strength of salmon gelatin with controlled molecular weight. Different letters show significant differences among the samples ($p<0.05$).

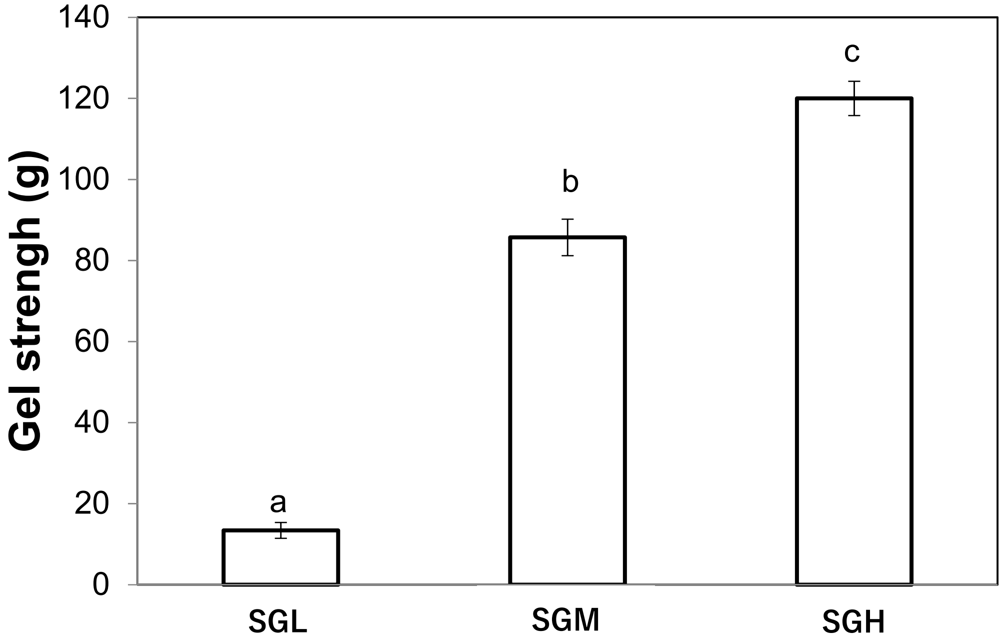

Figure 3.SM. Viscoelastic parameter Tan δ of salmon gelatin with controlled molecular weight tested by frequency sweep.

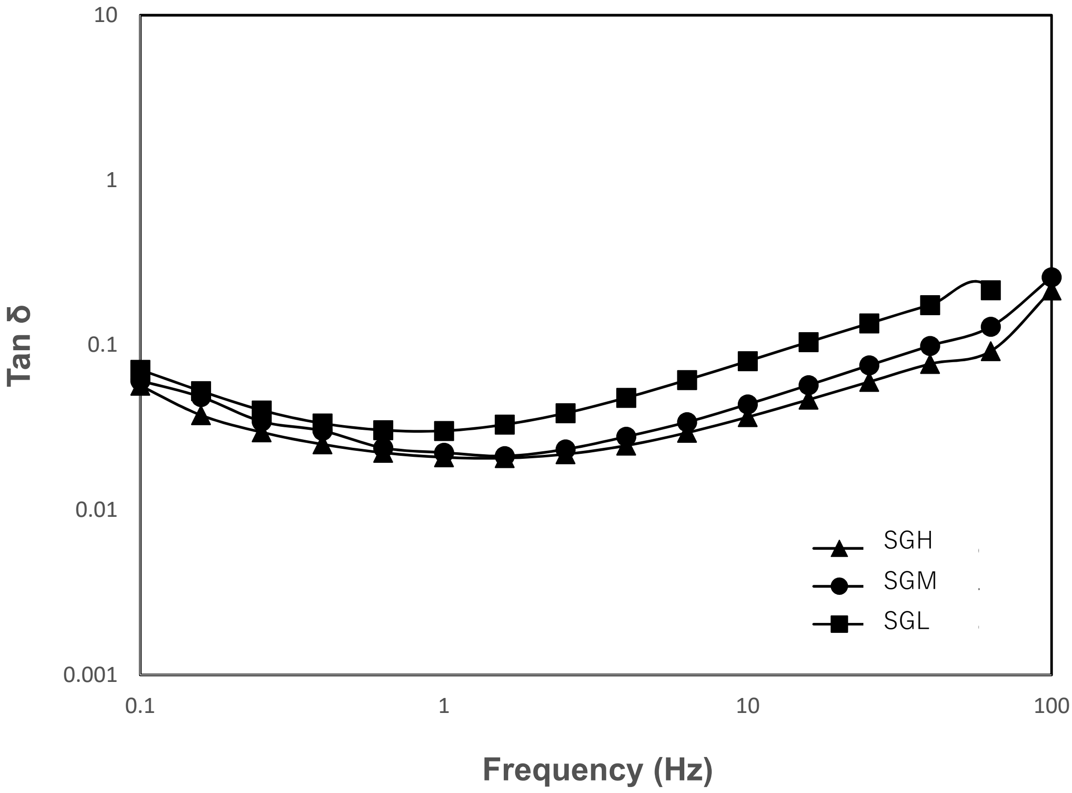

Supplement: Supplementary file 1 [file polymers-12-01587-s001.pdf]
